# Different Expression Patterns and Functions of Acetylated and Unacetylated Klf5 in the Proliferation and Differentiation of Prostatic Epithelial Cells

**DOI:** 10.1371/journal.pone.0065538

**Published:** 2013-06-05

**Authors:** Changsheng Xing, Xiaoying Fu, Xiaodong Sun, Peng Guo, Mei Li, Jin-Tang Dong

**Affiliations:** 1 Department of Genetics and Cell Biology, College of Life Sciences, Nankai University, Tianjin, China; 2 Department of Hematology and Medical Oncology, Emory Winship Cancer Institute, Emory University School of Medicine, Atlanta, Georgia, United States of America; 3 Department of Pathology, Tianjin University of Traditional Chinese Medicine, Tianjin, China; The University of Hong Kong, Hong Kong

## Abstract

KLF5 is a basic transcription factor that regulates multiple biological processes. While it was identified as a putative tumor suppressor in prostate cancer, likely due to its function as an effector of TGF-β in the inhibition of cell proliferation, KLF5 is unacetylated and promotes cell proliferation in the absence of TGF-β. In this study, we evaluated the expression and function of KLF5 in prostatic epithelial homeostasis and tumorigenesis using mouse prostates and human prostate epithelial cells in 3-D culture. Histological and molecular analyses demonstrated that unacetylated-Klf5 was expressed in basal or undifferentiated cells, whereas acetylated-Klf5 was expressed primarily in luminal and/or differentiated cells. Androgen depletion via castration increased both the level of Klf5 expression and the number of Klf5-positive cells in the remaining prostate. Functionally, knockdown of KLF5 in the human RWPE-1 prostate cell line decreased the number of spheres formed in 3-D culture. In addition, knockout of *Klf5* in prostate epithelial cells, mediated by probasin promoter-driven Cre expression, did not cause neoplasia but promoted cell proliferation and induced hyperplasia when one *Klf5* allele was knocked out. Knockout of both *Klf5* alleles however, caused apoptosis rather than cell proliferation in the epithelium. In castrated mice, knockout of *Klf5* resulted in more severe shrinkage of the prostate. These results suggest that KLF5 plays a role in the proliferation and differentiation of prostatic epithelial cells, yet loss of *KLF5* alone is insufficient to induce malignant transformation in epithelial cells.

## Introduction

Krüppel-like factor 5 (KLF5, also known as BTEB2 or IKLF) is a basic transcription factor that is widely expressed in different types of tissues [1], [2]. It belongs to the KLF family, which is structurally characterized by three zinc-finger domains at the C-terminus [2]–[4]. As a transcription factor, KLF5 directly binds to the promoters of many genes to regulate gene transcription in different biological processes including cell proliferation, survival and differentiation [2], [5]–[7]. Notably, KLF5 is necessary for cell proliferation and knockout of both *Klf5* alleles is embryonic lethal [8]. KLF5 is typically pro-proliferative in non-transformed epithelial cells, which are most likely equivalent to progenitor cells. For example, KLF5 is highly expressed in rapidly proliferating basal cells of the normal intestine, but its expression is reduced in mature and differentiated cells; and loss of Klf5 in mouse intestine significantly reduced the size of villi [9]. On the other hand, KLF5 inhibits the proliferation of cancer cells including those from the esophagus, prostate, breast and epidermis [10]–[13]. The bifunctional effects of KLF5 on cell proliferation could be due to post-translational modification under different cell contexts, as the pro-proliferative KLF5 becomes acetylated to inhibit cell proliferation upon the activation of TGF-β signaling, and interruption of its acetylation prevents its functional reversal in the proliferation of epithelial cells [13], [14].

Prostate cancer is the second most common malignancy and the second leading cause of cancer death in American men. It is generally recognized that molecular abnormalities that enhance cell proliferation and/or interfere with cell differentiation transform a normal epithelial cell to a cancer cell, yet the molecular events that underlie normal epithelial homeostasis and malignant transformation are still not well understood. The *KLF5* gene centers a common region of deletion at 13q21 in human cancers including prostate cancer, suggesting a tumor suppressor function for KLF5 [10], [11], [15]. Deletion of *KLF5* in human cancers is almost exclusively hemizygous [10], [11], which reduces *KLF5* transcription by half because *KLF5* is haploinsufficient [8]. In addition, ectopic expression of *KLF5* in prostate cancer cells inhibits cell proliferation [11], [13] and suppresses tumorigenesis in a xenograft model [16]. These findings suggest that KLF5 plays a tumor suppressor role in prostate cancer, yet such a role has not been examined in a mouse model with the deletion of *Klf5*.

Androgen and androgen receptor (AR) are essential for the normal development and function of the prostate. Although AR signaling primarily induces differentiation in normal prostates, it promotes the proliferation of prostate cancer cells. In the AR-positive LNCaP prostate cancer cell line, which has lost one copy of the *KLF5* genome [11], KLF5 appears to be a direct target and functional co-factor of AR in transcriptional regulation of AR target genes [17]. It is thus possible that KLF5 plays a role in prostate homeostasis in the context of AR signaling, which has not been tested.

In this study, we evaluated the expression patterns of Klf5 in adult mouse prostates, with and without androgen ablation. KLF5 expression was also examined in an *in vitro* model of human prostatic epithelial differentiation. We also generated a floxed-Klf5 mouse strain and knocked out *Klf5* in the prostate by crossing these mice to the PB-Cre4 mice, in which the *Cre* gene is expressed under the probasin promoter [18]. While localized to the nucleus of epithelial cells, acetylated Klf5 (Ac-Klf5) was primarily expressed in luminal and/or differentiated cells but unacetylated Klf5 (unAc-Klf5) was exclusively expressed in basal or undifferentiated cells. Klf5 expression was increased in castration-resistant prostate epithelial cells, and knockout of Klf5 resulted in more severe shrinkage of the prostate caused by castration. Deletion of *Klf5* was insufficient to initiate neoplasia, although deletion of one *Klf5* allele promoted cell proliferation and caused hyperplasia. Deletion of both *Klf5* alleles induced apoptosis. These results suggest that Klf5 plays a role in the homeostasis of prostate epithelial cells, yet deletion of *Klf5* alone is insufficient for neoplasia induction.

## Materials and Methods

### Ethics Statement

Mice used in these studies were housed at the Division of Animal Resources (DAR) facility at Emory University and handled by DAR stuff. All mice were closely monitored and humanely euthanized. All experimental procedures involving animals were approved by the Institutional Animal Care and Use Committee (IACUC Protocol No. 2001137).

### Mouse Strains

The floxed Klf5 mouse strain was generated by Ozgene Pty Ltd (Bentley DC, WA, Australia) by inserting two *loxP* sites into the mouse *Klf5* genome to flank the DNA fragment of exon 2, intron 2 and exon 3. The PB-Cre4 transgenic mice [18] were obtained from the NCI Mouse Models of Human Cancers Consortium (MMHCC, Frederick, MD, Cat#: 01XF5).

### Mouse Genotyping

All mice were toe-clipped and tailed at the age of 10–14 days for marking and genotyping, respectively. Tail tissues were processed and PCR-based genotyping was conducted as previously described [19]. Primer sequences and the sizes of PCR products are listed in Table 1.

**Table 1:** List of PCR primer sequences used in the generation of hybridization probes, genotyping and RT-PCR.

Description	Forward/reverse	
*Hybridization probes*		
**5′**	ACAAGAGCCATGAGTGCTTTTCCG/CTTTTAGAACATCAGGGATGCCACC	
**3′**	TGTATAGGAAGGAGCAAGGGACCAC/TCGTTTTCATGCACAGGGTGATG	
**En**	ATTTCTTGGGGTCTGCCAGTGTCG/CAGTCATCTTGCTATCTCCTGAGCG	
*Genotyping*		
***Klf5^wt^***	ACAGATTTGAGGCAGTTTGGC/GGGCCAACTCCTAAGTGTTGC	
***Klf5^mut^***	ACAGATTTGAGGCAGTTTGGC/CAAATCCTGCCCTCACCCTG	
***Cre***	CGGTCGATGCAACGAGTGAT/CCACCGTCAGTACGTGAGAT	
***Il-2***	CTAGGCCACAGAATTGAAAGATCT/GTAGGTGGAAATTCTAGCATCATCC	
*Real-Time PCR*		
***Klf5***	ACTGCCCTCGGAGGAGCTGG/ATGCTCTGAAATTATCGGAACTG	
***Gapdh***	CCAGCCTCGTCCCGTAGACA/GCCGTTGAATTTGCCGTGAG	

Nine-week-old male mice (*Cre^+^/Klf5^flox/wt^*) were tested for knockout efficiency. Freshly dissected organs were washed in PBS to remove blood, and incubated overnight at 56°C with tissue lysis buffer (100 mM Tris-HCl, 5 mM EDTA, 200 mM NaCl, 0.2% SDS, 500 µg/ml proteinase K, pH 8.0). Using diluted tissue lysates as templates, PCR with primers F and R2 was performed to determine the occurrence of deletion in different organs.

### Histopathology

Mice were sacrificed at various time points (postnatal 6, 12, 18 and 24 months), the mixture of prostate, urethra, bladder and surrounding connective tissues was isolated, and the prostate dissected from the mixture in PBS. Wet weights of prostates were measured immediately, and the data was analyzed by using the GraphPad Prism 5 software (GraphPad Software, Inc. La Jolla, CA).

Tissues for histopathological analysis were fixed in neutral buffered formalin overnight, embedded in paraffin, sectioned at 5-µm, and stained with hematoxylin and eosin (H&E). H&E slides were then examined on a blinded basis according to previously published criteria [20].

### Castration of Mice

Male mice were anaesthetized with a mixture of ketamine (40 mg/kg b.w.), xylazine (6.7 mg/kg b.w.) and buprenorphine (0.05 mg/kg b.w.) by intraperitoneal injection (i. p.) prior to surgery. A ventral midline incision was made in the scrotum to expose the testis. The spermatic artery was clamped and ligated, and the testis and epididymis were removed. The body wall and skin were closed with poliglecaprone suture (size 5-0, Monocryl. Ethicon, Somerville, NJ) in an interrupted pattern. Post-surgical mice were closely observed and returned to their cage after full recovery from anesthesia. For prostate regeneration after castration, testosterone pellets (Innovative Research of America, Sarasota, FL. Cat#: A-151) were subcutaneously implanted and placebo pellets (Cat#: C-111) were used as controls.

### RNA Extraction and Real-time PCR

To test the knockout efficiency at the RNA level, freshly dissected whole prostates were immediately immersed into the RNAlater reagent (Qiagen, Valencia, CA) for RNA stabilization. After overnight storage at 4°C, total RNA was isolated using the RNeasy Mini Kit (Qiagen) following the manufacturer’s instruction. First-strand cDNA was synthesized using the iScript cDNA synthesis kit (Bio-Rad, Hercules, CA).

Real-time RT-PCR was performed with the SYBR Premix Ex Taq reagent (Takara, Otsu, Shiga, Japan) with an ABI Prism 7500 Real-time PCR System (Applied Biosystems, Foster City, CA). The 2^(-ΔΔCt)^ method was used to calculate the relative expression level, with the expression of *Gapdh* as the internal control. Primer sequences for mouse *Klf5* and *Gapdh* are listed in Table 1.

### Immunohistochemistry (IHC) and Immunofluorescence (IF) Staining

For IHC staining, tissue sections were deparaffinized in xylene, rehydrated in graded ethanol solutions, and washed in tap water. Antigen retrieval was achieved by boiling the slides in a pressure cooker for 3 min in a citrated buffer (10 mM trisodium citrate, pH 6.0). After 10 min treatment with 3% H_2_O_2_, tissue sections were blocked with 5% normal goat serum in Tris-buffered saline with 0.1% Tween-20 (TBST) for 1 hour at room temperature, and incubated first with primary antibodies at 4°C overnight and then with EnVision Polymer-HRP secondary antibodies (Dako, Glostrup, Denmark) at room temperature for 30 min. After the application of DAB-chromogen, tissue sections were stained with hematoxylin, dehydrated, and mounted. The slides were then scanned with a Hamamatsu NanoZoomer scanner (Hamamatsu Corporation, Bridgewater, NJ), and cell numbers were counted to determine the percentage of positively stained cells for each protein.

For IF staining, tissue sections were incubated with the blocking solution (10% normal goat serum and 1% BSA in PBS) for 1 hour at room temperature after deparaffinization, rehydration and antigen retrieval. After incubation with primary antibodies at 4°C overnight and secondary antibodies (Alexa Fluor Dyes, Invitrogen, Carlsbad, CA) at room temperature for 30 min, tissue sections were treated with 4′,6-Diamidino-2-Phenylindole (DAPI) for nuclear staining, and mounted with anti-photobleaching mounting medium. Pictures were taken with the Zeiss Axioplan 2 Widefield Microscope (Carl Zeiss Microscopy, Thornwood, NY) with multi-channels.

Primary antibodies used in this study are listed in Table 2.

**Table 2:** List of antibodies used and related information.

Antigen	Supplier	IgG Type	Dilution
**KLF5**	Generated by Dong Lab	Rabbit IgG	IHC: 1∶6000, IF: 1∶200
**unAc-KLF5**	Generated by Dong Lab	Rabbit IgG	IHC: 1∶1000, IF: 1∶200
**Ac-KLF5**	Generated by Dong Lab	Rabbit IgG	IHC: 1∶1000, IF: 1∶200
**CK5**	Covance #PRB-160P	Rabbit IgG	IF: 1∶200
**CK14**	Thermo Scientific #MS-115-P0	Mouse IgG3	IF: 1∶200
**AR**	Upstate (Millipore) #06–680	Rabbit IgG	IF: 1∶200
**Ki67**	Thermo Scientific #RM-9106-S	Rabbit IgG	IHC: 1∶200
**Cleaved Caspase-3**	Cell Signaling #9661	Rabbit IgG	IHC: 1∶500

### Cell Culture and 3-D-culture

The RWPE-1 cell-line was purchased from the American Type Culture Collection (ATCC, Manassas, VA). Normal culture of RWPE-1 cells required the K-SFM medium (Invitrogen, Cat#: 17005–042) containing 0.2 ng/ml epidermal growth factor (EGF) and 25 mg/ml bovine pituitary extract (BPE). For 3-D culture, 50 µl/well matrigel mix (BD Bioscience, San Jose, CA) was firstly spread over the surface of 8-well chamber slides (Lab-Tek, Electron Microscopy Sciences, Hatfield, PA), which were left at 37°C for 1 hour to gel. Six-thousand cells were then seeded onto each well in 500 µl full medium including 2% (v/v) matrigel. Culture medium was replaced every three days, and spheres of well-differentiated cells could be observed after two weeks in 3-D-culture [21]. The siRNA for *KLF5* (si-KLF5) has been described previously [22], and the siRNA for luciferase (si-Luc) [22] was used as a control.

### Western Blotting

As described previously [23], cells were pre-treated with 20 µM MG132 for 4 hours, and collected with modified RIPA buffer (50 mM Tris/HCl, pH 8.0, 150 mM NaCl, 1% Nonidet P40, 0.5% sodium deoxycholate, 0.1% SDS, and 1% proteinase inhibitor I cocktail from Sigma) for Western blot analysis. Protein lysates were boiled with 2×SDS loading buffer (Bio-Rad) and subjected to Western blot analysis. The primary antibodies used in this study included KLF5 anti-serum (1∶1000 dilution) and anti-actin polyclonal antibody (Sigma-Aldrich, St. Louis, MO, Cat#: A2066, 1∶1500 dilution).

### TUNEL Assay

TumorTACS™ *In Situ* Apoptosis Detection Kit (Trevigen, Gaithersburg, MD, Cat#: 4815-30-K) was used for the TUNEL assay. Formalin fixed prostate sections from 18-month-old mice were stained following the manufacturer’s instruction. The slides were then scanned, and the total number of cells in the entire anterior prostate was counted to determine the percentage of apoptotic cells in each group.

## Results

### Expression of Different Forms of Klf5 in Mouse Prostates

**Figure 1:**
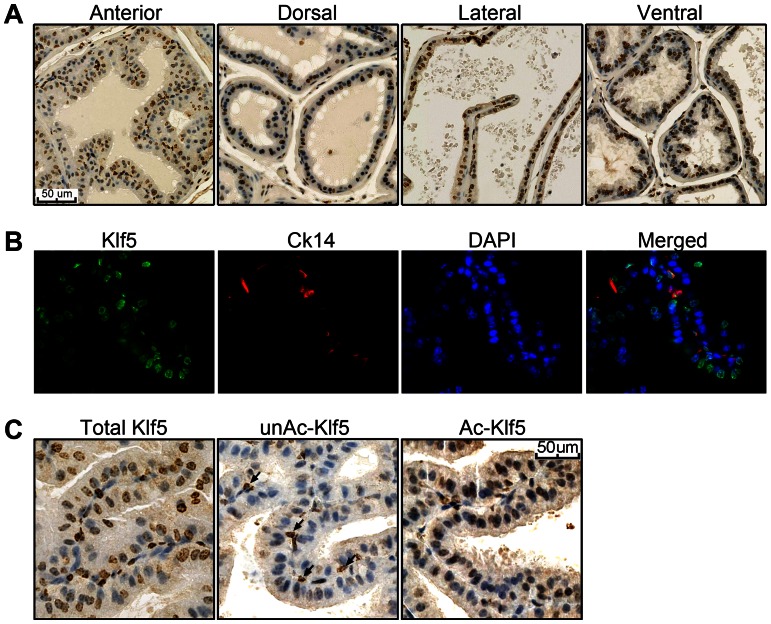
Expression of Klf5 in mouse prostate epithelial cells. A. Detection of total Klf5 by IHC staining with antisera against KLF5 protein in the four lobes of mouse prostates dissected from 24-week-old wildtype mice. The number of Klf5-positive cells was counted and the ratio to total number of cells was calculated and described in the text (n = 2). B. Double IF staining of Klf5 and Ck14 in adult anterior prostate of the mouse (Magnification: 200X). C. Detection of Ac-Klf5 and unAc-Klf5 in the adult anterior prostate. Arrows point to unAc-Klf5-expressing basal cells.

We first examined the expression of total Klf5, acetylated Klf5 (Ac-Klf5) and unacetylated Klf5 (unAc-Klf5) in adult mouse prostates with our previously generated antisera [24]. IHC staining demonstrated that the Klf5 protein was widely expressed in epithelial cells of all four lobes of mouse prostates (Fig. 1A). As expected for a transcription factor, Klf5 was mostly localized in the nucleus. Whereas both luminal and basal cells expressed Klf5, basal cells generally had more intense staining than luminal cells. In addition, almost all basal cells stained positive for Klf5 while not all luminal cells were positive. Double IF staining with Klf5 antiserum and basal cell marker Ck14 further showed that Klf5 was expressed in all basal cells but not all luminal cells of prostates (Fig. 1B). The rates of Klf5 positivity among luminal cells were 57.1% in anterior prostate (2436 out of 4268 cells), 61.3% in dorsal prostate (2878 out of 4695 cells), 61.0% in lateral prostate (731 out of 1199 cells), and 69.4% in ventral prostate (1209 out of 1742 cells), respectively.

Antibodies against Ac-Klf5 and unAc-Klf5 [14] were also used to determine the acetylation status of Klf5 in mouse prostates, because Ac-Klf5 and unAc-Klf5 have different functions in cell proliferation [13]. IHC staining showed that unAc-Klf5 was strictly located in basal cells of epithelia, whereas Ac-Klf5 was mainly expressed in luminal cells and a small number of basal cells (Fig. 1C). These results suggest that Ac-Klf5 and unAc-Klf5 have distinct functions in luminal and basal cells, and that unAc-Klf5 is more relevant to cell proliferation while Ac-Klf5 more relevant to cell differentiation.

### Expression and Function of KLF5 in Spheres of Cultured Human Prostate Epithelial Cells

**Figure 2:**
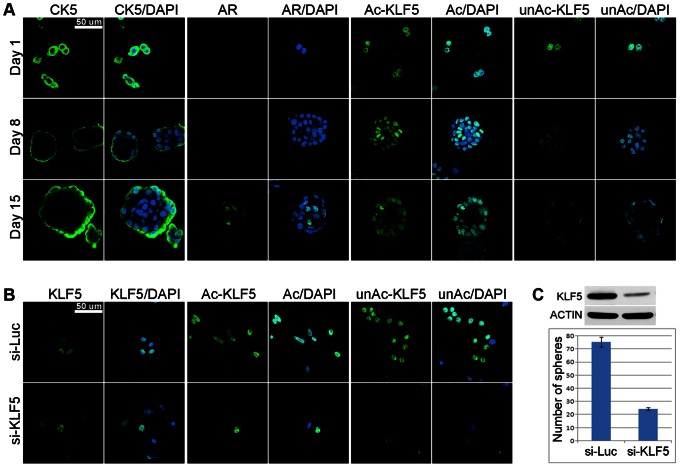
Expression of KLF5 in and its effect on sphere formation in RWPE-1 cells. A. Detection of Ac-KLF5 and unAc-KLF5 in RWPE-1 spheres, along with marker CK5 for undifferentiated cells and AR for differentiated cells. B. Detection of total KLF5, Ac-KLF5 and unAc-KLF5 by IF staining in 3-D-cultured RWPE-1 cells (Day 1 in matrigel) with the knockdown of *KLF5*. C. Confirmation of *KLF5* knockdown (upper panel) by Western blotting with antisera against KLF5 and the decrease in the number of spheres by the knockdown of *KLF5*. The same number of cells (6000) was seeded onto each chamber. After two-weeks of 3-D culture, the average number of spheres from 8 chambers per group was calculated.

Three-dimensional (3-D) culture of non-malignant prostatic epithelial cells can recapitulate acinar morphogenesis as the acini in 3-D culture appear highly representative of normal human prostate glandular structures, and thus have become useful for the investigation of prostate development [25]. To further test the function of KLF5 in prostatic epithelial differentiation, RWPE-1 cells were cultured in matrigel to form spheres, and IF staining was used to measure the distribution of different proteins. CK5, a marker of undifferentiated cells, was expressed in all RWPE-1 cells on the first day of culture in matrigel, but its expression was restricted to the outer layer of spheres after one to two weeks of culture (Fig. 2A). On the other hand, a marker of differentiated cells, androgen receptor (AR), was undetectable in cells from day 1 to day 8 but was highly expressed in the inner layer of spheres on day 15 (Fig. 2A). Consistent with previous studies showing the absence of AR in the 2-D culture of RWPE1 cells [21] but the induction of AR in 3-D culture of similar prostatic epithelial cells [26], these results suggest that spheres of RWPE-1 cells have fully differentiated at day 15. In addition, these results suggest that spheres have undifferentiated cells in the outer layer and differentiated cells in the inner layer, consistent with previous studies [25], [26]. IF staining showed that both Ac-KLF5 and unAc-KLF5 were expressed in all cells at day 1 and day 8, but at day 15, unAc-KLF5 was detected only in the undifferentiated outer layer of cells, while Ac-KLF5 was detected in both outer and inner layers (Fig. 2A), which is consistent with their distribution in mouse prostates.

To determine whether the loss of KLF5 affects the differentiation of RWPE-1 cells, *KLF5* was knocked down by RNAi before cells were seeded into matrigel. As indicated by IF staining with KLF5 antibodies, there were still a certain number of cells that had detectable KLF5 expression at day 1 in matrigel (Fig. 2B), although the level of total KLF5 decreased sharply after siKLF5 treatment (Fig. 2C). Knockdown of *KLF5* decreased the number of spheres by two thirds (Fig. 2C), indicating the necessity of KLF5 for sphere formation. This decrease could be due to the compromised function of KLF5 in either cellular differentiation induced by Ac-KLF5 or cellular proliferation mediated by unAc-KLF5. On the other hand, the size or structure of spheres as well as KLF5’s expression and localization did not change in the spheres that formed in the *KLF5* knockdown group (data not shown). Nor was the expression of AR, CK5 or KLF5 altered (data not shown), suggesting that those cells that formed spheres did not have efficient or maintained knockdown of *KLF5*.

### Androgen Ablation Altered the Expression and Distribution of Klf5

**Figure 3:**
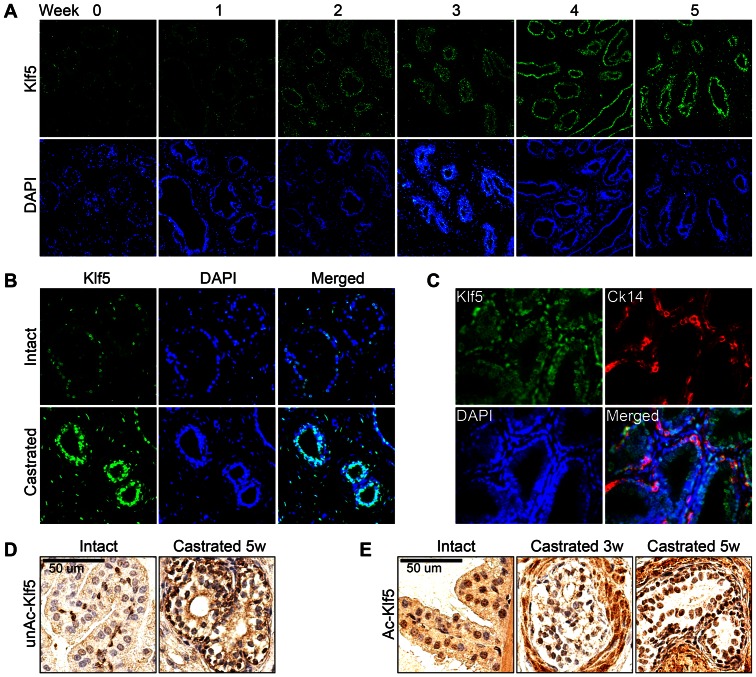
Androgen ablation increases Klf5 expression and the number of Klf5-expressing cells in mouse prostates. A. Detection of Klf5 by IF staining with total Klf5 antibody in the dorsal prostate at different times after castration (Magnification: 100X, n = 3 for each time point). B. Higher magnification (200X) of cells stained for Klf5 and DAPI in normal prostates and prostates undergoing androgen ablation for 5 weeks. C. Double IF staining of Klf5 and basal cell marker Ck14 in prostates after 5 weeks of castration (Magnification: 400X). D. IHC staining of unAc-Klf5 in intact and androgen-depleted prostates (5 weeks). E. IHC staining of Ac-Klf5 in intact and androgen-depleted prostates (3 and 5 weeks).

Androgen and androgen receptor (AR) signaling is essential for the development and maintenance of normal prostates, and a previous study suggests that KLF5 is directly regulated by AR signaling [17]. To investigate whether AR signaling regulates Klf5 *in vivo*, we performed castration in 12-week-old wildtype mice to deplete androgen, and detected Klf5 expression. IF staining with anti-Klf5 antiserum showed that the expression of total Klf5 was gradually increased from week 1 to week 5 after castration (Fig. 3A), and that the percentage of Klf5-positive cells also increased dramatically (Fig. 3B). After castration, the prostate undergoes severe shrinkage due to massive apoptosis in luminal cells, whereas the basal cells are enriched after the death of luminal cells. To evaluate Klf5 expression in different types of cells, we performed double IF staining with anti-Klf5 antiserum and anti-Ck14 antibody. Consistent with the findings from sham-castrated mice, Klf5 was expressed in all basal cells after castration. In luminal cells that survived apoptosis after castration, the percentage of Klf5-positive cells increased from 61.3% to 96.8% (211 out of 218 cells), suggesting a role for Klf5 in the survival of luminal cells after castration (Fig. 3C).

To determine the acetylation status of Klf5 in prostates after castration, the expression of unAc-Klf5 and Ac-Klf5 was examined by IHC staining. UnAc-Klf5 was exclusively expressed in the surrounding basal cells with a uniform staining intensity (Fig. 3D), which is similar to the expression of unAc-Klf5 in intact mice. The expression of Ac-Klf5 was detected in more luminal cells, and the percentage of Ac-Klf5-positive luminal cells reached 98% at 5 weeks post castration, which is similar to the expression of total Klf5 in luminal cells (Fig. 3E).

### Generation of the Floxed-Klf5 Mouse Strain

To further evaluate the function of Klf5 *in vivo*, we generated a floxed-Klf5 mouse strain. In this strain, one *loxP* site was inserted upstream of exon 2 and the other downstream of exon 3, which enabled the excision of exon 2, intron 2 and exon 3 by the Cre recombinase, leading to early termination of Klf5 translation and the truncation of 78% of the Klf5 protein, including all zinc-finger domains at the C-terminus.

**Figure 4:**
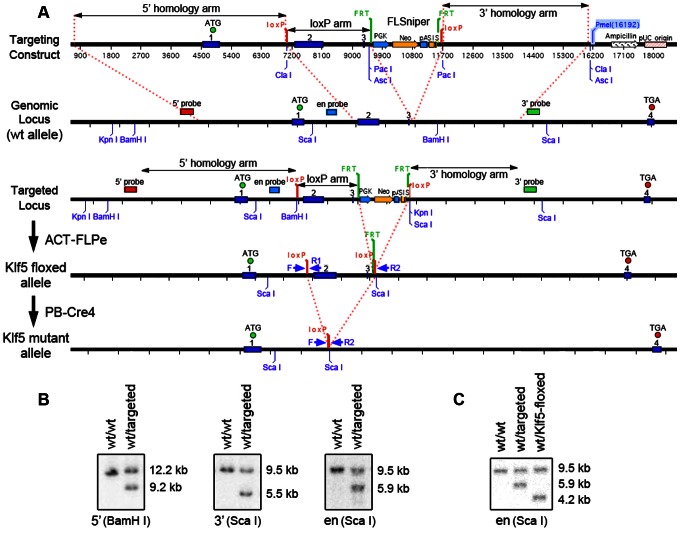
Generation of floxed-Klf5 mouse strain. A. Strategy for the generation of floxed-Klf5 mice, including the design of targeting construct as compared to the endogenous *Klf5* locus, the generation of targeted locus by homologous recombination, and the production of floxed *Klf5* allele and the mutant allele by a series of crossing. B. Confirmation of successful targeting in embryonic stem cells by Southern blotting using probes for the 5′ arm, 3′ arm and endogenous (en) fragment. C. Confirmation of the floxed allele by Southern blotting with the endogenous probe in mice after breeding.

The mouse strain was generated at the Ozgene facility. Briefly, the 3′ homology arm (4.4 kb), *loxP* arm (2.5 kb) and 5′ homology arm (6.3 kb) were amplified from C57BL/6 genomic DNA, and cloned into the FLSniper plasmid at sites *Pac*I, *Asc*I and *Cla*I respectively (Fig. 4A). After digestion with the *Pme*I restriction enzyme, the linearized plasmid was electroporated into mouse embryonic stem (ES) cells. After selection, positive ES clones were identified by Southern hybridization with three probes: 5′ probe, 3′ probe and endogenous probe (en probe) (Fig. 4B). PCR primers used for cloning these probes are listed in Table 1. Appropriately targeted ES cells were microinjected into blastocysts and implanted into recipient female mice. Extensively chimeric mice were mated to C57BL/6 mice, and F1 heterozygous mice were crossed with ACT-FLPe mice, which expressed the FLP enzyme and enabled the excision of the FLSniper cassette from genome in offsprings (Fig. 4A). After confirmation of *Flp* deletion by another Southern hybridization with the endogenous probe (Fig. 4C), mice were bred with C57BL/6 again to generate the floxed-Klf5 strain. Mice homozygous for the floxed-Klf5 allele were viable and fertile and did not display any gross physical or behavioral abnormalities.

### Prostate-specific Deletion of *Klf5* in Mice

**Figure 5:**
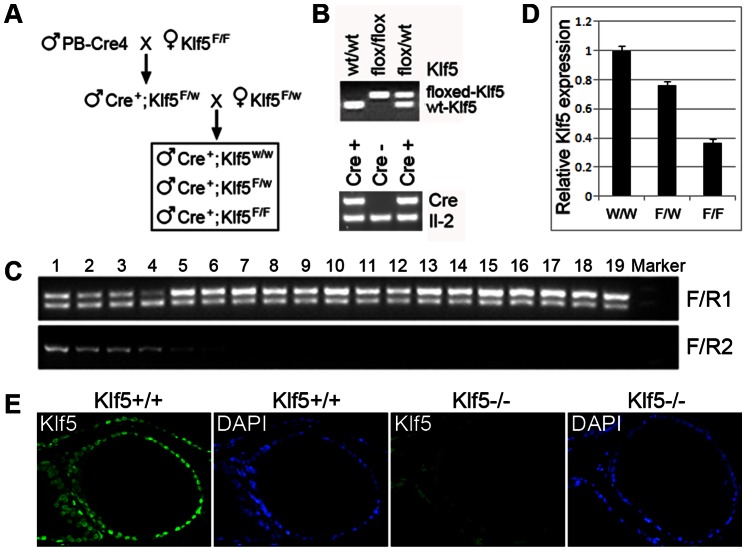
Confirmation of *Klf5* deletion mediated by the expression of Cre in mouse prostates. A. Breeding strategy for the production of mice with the three desired genotypes of *Klf5*. B. PCR-based genotyping of *Klf5*, floxed *Klf5*, and *PB-Cre*, with *Il-2* as a control for the detection of *Cre*. C. Detection of *Klf5* knockout in different tissues from 9-week-old heterozygous mice. Primers F and R1 amplify the wildtype allele while primers F and R2 amplify the knockout allele. Tissues in the lanes are: 1, anterior prostate; 2, dorsal prostate; 3, lateral prostate; 4, ventral prostate; 5, seminal vesicle; 6, urethra; 7, testis; 8, bladder; 9, heart; 10, brain; 11, lung; 12, liver; 13, stomach; 14, kidney; 15, spleen; 16, small intestine; 17, colon; 18, salivary gland; and 19, tail. D. Detection of *Klf5* mRNA by real-time RT-PCR in whole prostates of 13-week-old mice with different deletion status of *Klf5* (w/w, wildtype; f/w, heterozygous deletion; f/f, homozygous deletion). E. Reduced expression of Klf5 protein by the knockout of *Klf5*, as detected by IF staining in prostates from 16-week-old mice (Magnification: 200X).

Floxed-Klf5 mice were crossed with PB-Cre4 mice, which express the Cre recombinase under the prostate-specific probasin promoter, to generate three desired *Klf5* genotypes with a positive *Cre* genotype: *Cre^+^/Klf5^wt/wt^*; *Cre^+^/Klf5^flox/wt^* and *Cre^+^/Klf5^flox/flox^* (Fig. 5A). PCR primers F and R1 were designed to genotype *Klf5* alleles (Fig. 4A), in which the floxed-Klf5 allele was 34 bp longer than the wildtype (wt) allele because of the insertion of a *loxP* site (Fig. 5B). Existence of the *Cre* gene was also confirmed by PCR, with endogenous *Il-2* as the positive control (Fig. 5B). Primers F and R2 were designed to detect the deletion of the *Klf5* allele, and could only amplify the mutant allele in a regular PCR (Fig. 4A). Using DNA from different organs of a 9-week-old mouse, PCR with primers F and R2 showed that knockout of *Klf5* occurred in each of the prostate lobes, seminal vesicle and urethra but not in other organs or tissues tested (Fig. 5C). Using total mRNA from prostates of mice with three different genotypes at the age of 13 weeks, real time PCR showed that *Klf5* mRNA decreased proportionally to the number of knockout alleles (Fig. 5D). The knockout efficiency at the protein level was also measured by immunofluorescent (IF) staining, which showed that homozygous deletion dramatically decreased Klf5 protein expression as early as 16 weeks of age (Fig. 5E). Deletion of one *Klf5* allele decreased Klf5 protein level but the decrease did not appear to be half of that in normal prostates (data not shown).

### Knockout of one *Klf5* Allele Induced Hyperplasia and Increased Cell Proliferation but Knockout of Both Alleles Caused Apoptosis

**Figure 6:**
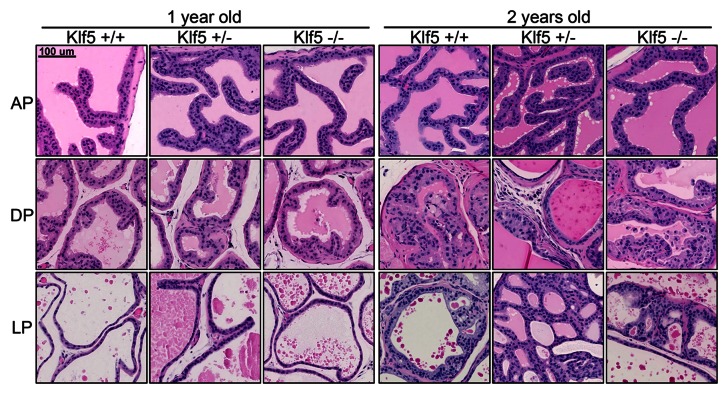
Morphologies of mouse prostates with different deletion status of *Klf5*. Three prostate lobes, including anterior prostate (AP), dorsal prostate (DP) and lateral prostate (LP), from two time points (one and two years) were examined (at each time point, n> = 5 for Klf5^+/+^ mice and n> = 9 for Klf5 knockout mice).

To evaluate the role of Klf5 in the proliferation, differentiation and tumorigenesis of mouse prostates, histological and molecular analyses were performed in mice with different *Klf5* deletion status and at various ages. At the age of one year, no mouse prostatic intraepithelial neoplasia or mPIN, a neoplastic alteration in mouse prostates, was detected in any of the knockout mice, although benign hyperplasia was widely detected (Fig. 6). Compared to mice with wildtype *Klf5* (*Cre^+^/Klf5^wt/wt^*) or null *Klf5* (*Cre^+^/Klf5^flox/flox^*), mice with the knockout of one *Klf5* allele (*Cre^+^/Klf5^flox/wt^*) had more severe hyperplasia, with thicker cell layers in the anterior prostate, dorsal prostate and lateral prostate (Fig. 6, left panel). *Klf5* deletion had no visible histopathologic effects in the ventral prostate, which has no anatomical counterpart in human prostate [27]. At the age of two years, mPIN lesions were detected in the dorsal and lateral prostates of wildtype mice, which is associated with aging and/or genetic background [28]. Knockout of *Klf5* did not increase the incidence of mPIN in the prostates, as the incidences in the dorsal prostate were 80% (4/5) in *Klf5^+/+^* mice, 71.4% (10/14) in *Klf5^+/−^* mice, and 77.8% (7/9) in *Klf5^−/−^* mice; and the incidences in the lateral prostate were 80% (4/5) in *Klf5^+/+^* mice, 45.5% (5/11) in *Klf5^+/−^* mice, and 77.8% (7/9) in *Klf5^−/−^* mice. Consistent with the results from 1 year old mice, more layers of atypical cells were observed in the heterozygous group than in the wildtype or homozygous group (Fig. 6, right panel). Notably, at both time points, mice with one *Klf5* allele tended to have more glandular infoldings in the anterior prostate (Fig. 6, S1) than either wildtype mice or *Klf5*-null mice. Furthermore, prostates with null *Klf5* had no obvious phenotypic differences from those with wildtype *Klf5*, which was more obvious when examining prostate acini at a lower magnification (Fig. S1). The weights of prostates were also measured at the time of isolation, and mice with hemizygous deletion of *Klf5* tended to have heavier prostates than those with wildtype *Klf5* or homozygous deletion (Fig. S2). The difference was not statistically significant though, which is likely due to smaller sample sizes and/or interindividual variation.

**Figure 7:**
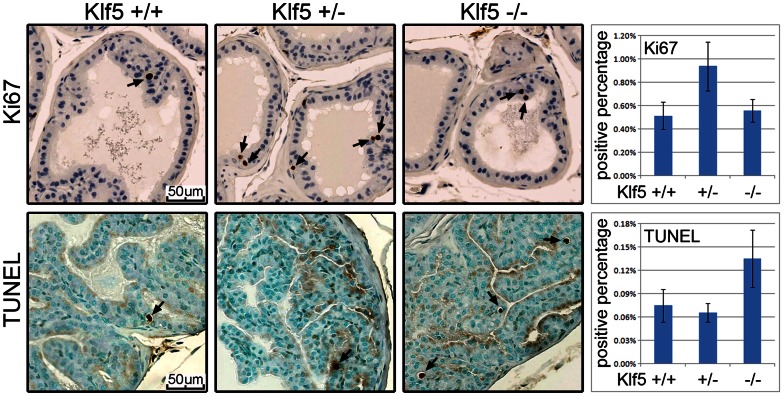
*Klf5*’s hemizygous deletion promotes cell proliferation and induces hyperplasia while its homozygous deletion causes apoptosis in the prostate. Shown in the upper panels is the IHC staining of Ki67 in 1-year-old dorsal prostates, and the ratio of Ki67-positive cells is significantly higher in the prostate with *Klf5* hemizygous deletion than those with wildtype *Klf5* or homozygous deletion of *Klf5* (upper right panel). The lower panels show apoptotic cells (indicated by arrows) as detected by TUNEL assay in the anterior prostates from 1.5-year-old mice, and the ratio of apoptotic cells in the homozygous deletion group is about double that in the wildtype or hemizygous deletion group (lower right panel). Three mice per genotype were counted.

We then analyzed the rate of cell proliferation and apoptosis in prostates with different status of *Klf5* deletion. Staining of the Ki67 proliferation marker showed that the cell proliferation rate was significantly higher in prostates with one *Klf5* allele than that in mice with wildtype *Klf5* or null *Klf5* (Fig. 7, upper panel). On the other hand, TUNEL assay showed that deletion of both *Klf5* alleles induced apoptosis whereas knockout of one *Klf5* allele did not have such an effect (Fig. 7, lower panel). Consistently, cleaved caspase-3 was detected in a small number of cells from *Klf5*-null prostates but not in cells from mice with wildtype *Klf5* or hemizygous deletion of *Klf5* (data not shown).

### Loss of Klf5 Promotes Castration-induced Shrinkage of the Prostate

After castration, the prostate shrinks due to massive apoptosis of luminal epithelial cells. The ratio of Klf5-positive cells increased in the surviving luminal cells (Fig. 3B, C), suggesting that Klf5 expression could enhance cell survival after castration. To further test this hypothesis, weights of freshly isolated prostates were measured in mice with different status of castration and *Klf5* deletion. Five weeks after castration, knockout of *Klf5* by either one allele or both alleles appeared to further decrease prostate weights (Fig. S2B). The difference in prostate weight between wildtype mice and *Klf5*-null mice was retained even after androgen was re-administered by subcutaneous implantation of androgen pellets for four days, although prostate weights in both groups increased when compared to mice without androgen administration (Fig. S2C). Two weeks after androgen administration, prostates were fully recovered to normal sizes, and the difference in prostate weight between the two groups disappeared (Fig. S2D). We noticed that the differences were not statistically significant, likely due to a smaller number of mice and wide interindividual differences among mice.

## Discussion

### Different Expression Patterns of Ac-Klf5 and unAc-Klf5 in Mouse Prostate

In this study, the expression pattern of Klf5 was detected in mouse prostate for the first time. Klf5 was widely expressed in the nucleus of both luminal and basal epithelial cells of all lobes of mouse prostates. Stronger staining was detected in 100% of basal cells, whereas the staining was not as strong in luminal cells and only half of the luminal cells were positive for Klf5 (Fig. 1). This observation is consistent with the recognition that KLF5 expression is usually higher in basal cells or undifferentiated cells and lower in luminal cells or differentiated cells in other tissues. Our previous study of KLF5 expression in different prostate cell lines have shown that the RNA level of KLF5 is higher in PZ-HPV-7, RWPE-1, DU 145, DU Pro and PC-3 cell lines [11], which are commonly considered as basal-originated cells [29]–[32]; while much lower in LNCaP, 22Rv1 and BRF-41T cell lines [11], which have luminal cell features [29]–[32].

The necessity of KLF5 in stem cell renewal and maintenance has been well established. For example, Klf5 activates the expression of self-renewal-promoting genes while inhibiting the expression of differentiation-related genes in mouse embryonic stem cells (ESCs) [33]. In addition, constitutive expression of Klf5 prevents the differentiation of ESCs [34] whereas depletion of Klf5 and other factors promotes ESC differentiation [35]. In the epidermis, overexpression of Klf5 causes hyperplasia of basal cells accompanied with the lack of mature skin [36]. The expression pattern of Klf5 in mouse prostate and prostate cancer cell lines suggests that Klf5 plays a similar role in the prostate, and is important in regulating the balance of proliferation and differentiation in prostate epithelial cells.

In mouse prostates, unAc-Klf5 was restricted to basal cells whereas Ac-Klf5 was mostly detected in luminal cells (Fig. 1). Consistent findings were obtained from an *in vitro* model of human prostate epithelial homeostasis – the 3-D culture of the RWPE-1 cell line, where unAc-KLF5 was detected only in the undifferentiated outer layer of spheres whereas Ac-KLF5 was detected in both outer and inner layers (Fig. 2). The differences on their localization suggest that unAc-Klf5 and Ac-Klf5 have distinct functions in prostate epithelia.

### UnAc-Klf5 May Function in the Proliferation of Prostate Epithelial Cells

Although TGF-β renders KLF5 an anti-proliferative factor by inducing its acetylation in cultured epithelial cells [13], [14], [22], KLF5 has long been recognized as a pro-proliferative factor in both epithelial cells and fibroblasts [2]. In our study, unAc-Klf5 was restricted to basal cells, whereas Ac-Klf5 was mainly expressed in luminal cells (Fig. 1). In addition, in androgen-depleted prostates, where basal cells are dramatically enriched and epithelial differentiation is interrupted [37], many more cells became positive for unAc-Klf5 staining while fewer were positive for Ac-Klf5 staining (Fig. 3). Taken together with a pro-proliferative function for unAc-KLF5 and anti-proliferative function for Ac-KLF5 in cultured cells *in vitro* [13], it is possible that unAc-Klf5 executes the pro-proliferative function of Klf5 in the proliferation of basal stem/progenitor cells. While we are testing this prediction using transgenic mice in which Klf5 is deficient in acetylation, we have found that unAc-KLF5 promotes the proliferation and tumorigenesis of prostate cancer cells (Li et al., manuscript in preparation).

Consistently, in the 3-D culture of human cells where unAc-KLF5 expressed in all the undifferentiated cells before seeded into matrigel, knockdown of KLF5 decreased the number of mature spheres (Fig. 2). One possible explanation is that loss of unAc-KLF5 decreased cell proliferation. We noticed that in the spheres that formed in the group treated with *KLF5* siRNA, the size or structure of spheres as well as the expression and localization of KLF5, AR, and CK5 did not change (data not shown), suggesting that cells that could form spheres did not have efficient knockdown of *KLF5*.

### Ac-Klf5 May Function in the Differentiation of Prostate Epithelial Cells

In testing the function of KLF5 in prostatic epithelial homeostasis, we found that knockout of one *Klf5* allele promoted cell proliferation and induced hyperplasia in luminal cells (Fig. 6, 7), indicating that Klf5 functions to restrain cell proliferation in luminal cells. Consistent with the findings that Ac-Klf5 rather than unAc-Klf5 is expressed in luminal cells (Fig. 1) and that acetylation of KLF5 is necessary for TGF-β to inhibit the proliferation of epithelial cells *in vitro* [13], the current study re-confirmed an anti-proliferation function of Ac-Klf5 in prostate epithelial cells.

The role of KLF5 in the differentiation of epithelial cells has been documented in other types of tissues, including bladder urothelium [38], intestinal epithelium [39], [40], and the lung [41]. Klf5 also plays a necessary role in the differentiation of adipocytes [42]. In the prostate, where luminal cells result from the differentiation of proliferating progenitor cells [43], Ac-Klf5 is proved to be expressed in both luminal cells and basal cells, and has an anti-proliferation function, whereas unAc-Klf5 is restricted to basal cells and has a pro-proliferation function. These results suggest that the acetylation of Klf5 may be relevant to the differentiation of the prostate epithelial cells. Taken together with the common recognition that the cytokine TGF-β is responsible for differentiation-induction [44], [45] and that the acetylation of KLF5 is not only induced by but also necessary for TGF-β to inhibit cell proliferation [13], we propose that the differentiation of prostate epithelial cells may start from the acetylation of Klf5 in progenitor cells, and complete after unAc-Klf5 disappears. Staining of keratins suggests that the differentiation of prostate epithelial cell is a multi-step process, and luminal epithelia consist of both well-differentiated cells and cells in intermediate state [46]. Ac-Klf5 may exist in luminal cells as an intermediate state, and vanish upon complete differentiation. Consistently, in the 3-D culture model where sphere formation represents the differentiation of proliferating epithelial cells [25], [47], RNAi-mediated knockdown of KLF5 decreased the number of spheres by two thirds (Fig. 2), possibly because of the impaired differentiation due to loss of Ac-KLF5.

Whether the acetylation of KLF5 is responsible for the function of Klf5 in epithelial differentiation cannot be concluded at this time. Currently we are in the process of generating a transgenic mouse model in which Klf5 is deficient in acetylation due to a mutation at its acetylation site, which will allow us to test the role of Ac-Klf5 in epithelial differentiation.

### Klf5 May Play a Role in the Function of Androgen Signaling in the Prostate

Androgen-AR signaling is essential for the proliferation, differentiation and maintenance of prostate epithelia and their normal functions [37]. Androgen depletion via castration causes the death of over 80% of luminal epithelial cells in the prostate [48], which is the principle for the commonly used androgen ablation therapy in the treatment of prostate cancer [49]. Combined with both the pro-proliferative and anti-proliferative functions of Klf5 in epithelial cells, it is possible that Klf5 is a functional effector of androgen-AR signaling in the homeostasis of prostatic epithelia. For example, when androgen signaling is activated to induce cell differentiation, Ac-Klf5 could become a cofactor for the AR transcriptional complex in the regulation of differentiation genes. In fact, KLF5 appears to be not only a direct target gene but also a functional co-factor of AR in the regulation of AR target genes in the AR-positive LNCaP prostate cancer cell line [17]. In the current study, we were unable to test the effect of Klf5 deficiency on the function of androgen signaling in cell differentiation because the promoter that drove the expression of Cre in the knockout of *Klf5* was from the probasin gene, which is a direct target gene of AR. If Ac-Klf5 rather than unAc-Klf5 is essential for cell differentiation as we predict, deficiency in the acetylation of Klf5 would interrupt androgen-induced cell differentiation in the prostate. This possibility is currently under test.

On the other hand, both the number of Klf5-expressing cells and Klf5 staining intensity were increased in those luminal cells that survived castration (Fig. 3C). There are at least two possible explanations for this. One is that androgen depletion attenuates the differentiation signal for cell differentiation, leading to the accumulation of basal-like, more rapidly proliferating cells, which have more intensive Klf5 staining as seen in basal cells *in vivo* and proliferating prostate epithelial cells *in vitro*. Indeed, the majority of the castration-surviving cells are also positive for the basal cell marker Ck14 (Fig. 3C), supporting such a possibility. Another explanation is that Klf5 promotes cell survival, and cells expressing higher levels of Klf5 survive better during castration-induced apoptosis. Previous studies have demonstrated a role of KLF5 in cell survival. For example, KLF5 binds to the promoter of the survivin gene to induce the expression of survivin in leukemia, which leads to increased cell survival [50]. In addition, knockdown of KLF5 in cancer cells increases their sensitivity to DNA damage and related cell death, which is associated with reduced BAD phosphorylation and downregulation of PIM1 [7].

### The Dose-dependent Manner of KLF5

Not only is the *KLF5* gene haploinsufficient [8], KLF5 protein is also sensitive to degradation mediated by the ubiquitin-proteasome pathway in epithelial cells [23], [24], [51], which indicates that the expression level of KLF5 is important for its proper function. Lack of more dramatic phenotypic alterations in prostates with the knockout of one *Klf5* allele could be due to a fine-tuned regulation of Klf5 expression. For example, downregulation of *Klf5* mRNA by the deletion of one allele could be compensated by slowed protein degradation. Supporting this possibility is that staining intensity in prostates with one *Klf5* allele was often indistinguishable from the staining in normal prostates (data not shown).

While hardly detectable in prostates with wildtype Klf5 or one knockout allele of Klf5, apoptosis was detected in 0.14% of luminal cells from *Klf5*-null prostates (Fig. 7), suggesting that complete loss of Klf5 is lethal to some luminal cells. This is consistent with the observations in hundreds of human prostate and other cancers, whereas hemizygous deletion at the *KLF5* locus occurs in as frequently as 40% or more of cases, homozygous deletion is hardly detectable [11], [52]. Apoptosis is a rapid process, and thus the number of apoptotic cells detected at a given time could be less than the actual number of dead cells. Furthermore, we noticed that staining of Klf5, although significantly weaker, was still detectable in a certain percentage of luminal cells that survived homozygous deletion of *Klf5* (Fig. 5E). Klf5 was detected in about 60% of luminal cells in normal prostates (Fig. 1). In addition, luminal cells are either differentiated and thus non-dividing or slowly dividing, so it is possible that after the deletion of *Klf5*, its protein degradation is decelerated and some Klf5 protein molecules remain in *Klf5*-null cells, preventing more extensive cell death. If complete loss of *Klf5* indeed causes cell death, its effect on cell differentiation can only be tested if cell death is prevented. We are currently testing this possibility by examining mouse prostates with the deletion of both *Klf5* and *Pten*, where deletion of *Pten* prevents cell death.

### Hemizygous Deletion of KLF5 and Tumorigenesis

Chromosomal deletion is a hallmark of tumor suppressor genes, and the locus spanning the *KLF5* gene, 13q21, is the second most frequently deleted chromosomal locus in over 70 types of human malignancies [15], [53]. In addition, *KLF5* was shown to center the deletion at 13q21 in human prostate cancer [11]. Therefore, *KLF5* was proposed to be a tumor suppressor gene [10], [11], even though it can transform fibroblasts and promote the proliferation of epithelial cells and tumorigenesis of some cancer cell lines [6], [54]–[56]. Recently, KLF5 was shown to suppress the tumorigenesis of prostate cancer cells in xenograft models [16], supporting a tumor suppressor function of KLF5. In the mouse prostate, although hemizygous deletion of *Klf5* increased cell proliferation and induced hyperplasia, which is consistent with a tumor suppressor activity, no neoplasia was detected (Fig. 6, 7). Lack of a neoplastic phenotype however, does not disapprove a tumor suppressor function of Klf5, because malignant transformation often requires multiple genetic alterations and knockout of *Klf5* alone is most likely insufficient for tumor induction. We are currently testing whether simultaneous deletion of *Klf5* and other genes that are frequently deleted in human prostate cancer, including *PTEN* at 10q23 and *NKX3-1* at 8p21, could induce tumorigenesis. Our preliminary data indicates that knockout of *Klf5* significantly promotes prostatic tumorigenesis induced by the deletion of *Pten* but has no additive effects with the knockout of *Nkx3-1* (Xing et al., manuscripts in preparation).

Although hemizygous deletion of *KLF5* is highly frequent in human prostate cancer, no homozygous deletion was detected in hundreds of human cancer specimens [10], [11], [52], in contrast to the deletion of two other frequently deleted loci – *NKX3-1* at 8p21 and *PTEN* at 10q23. It is possible that deletion of both *KLF5* alleles is lethal to a cell, as indicated by the finding that deletion of both *Klf5* alleles in mouse prostates caused significant apoptosis but did not induce detectable cell proliferation (Fig. 7). In addition, homozygous deletion of *Klf5* also induced greater shrinkage of the prostate caused by castration (Fig. S2B). Therefore, hemizygous deletion, which causes haploinsufficiency of *Klf5* [8], could be a common tumor promoting event.

In summary, we found that Klf5 was highly expressed in the nucleus of epithelial cells from both mouse prostates and 3-D culture of human prostatic epithelial cells. In addition, unAc-Klf5 was restricted to undifferentiated basal cells whereas Ac-Klf5 was expressed primarily in luminal cells and some basal cells. Androgen ablation via castration led to an increase in both Klf5 expression level and the number of Klf5-positive cells in the remaining prostate epithelia. Knockout of *Klf5* in mouse prostates promoted cell proliferation when the knockout occurred at one allele, although no mPIN was induced. Knockout of both *Klf5* alleles induced apoptosis in epithelial cells. Castration in *Klf5*-null prostates caused more severe shrinkage of the organ upon castration. Taken together with previous findings of context-dependent functions of KLF5 in cell proliferation and frequent deletion of *KLF5* in human prostate cancer, these results suggest that KLF5 plays an important role in the proliferation, differentiation and tumorigenesis of prostate epithelial cells.

## Supporting Information

Figure S1 **Lower magnification (40X) views of anterior and lateral prostates with different status of** ***Klf5*** **deletion.** Architectural differences are more obvious in these two lobes of the prostate at lower magnification. Prostates from 2-year-old mice with different deletion status of *Klf5* were subjected to H&E staining and histological analysis.
(TIF)

Figure S2 **Effects of androgen ablation on prostate weights with different status of** ***Klf5*** **deletion.** A. Weights of prostates with wildtype *Klf5* (+/+), hemizygous deletion of *Klf5*, or homozygous deletion of *Klf5* from 21-month-old intact mice without the ablation of androgen (n> = 5). B. Weights of prostates after 5 weeks of androgen ablation from 21-month-old mice (n> = 5). C & D. Weights of prostates from adult mice that underwent castration for 5 weeks and subsequent androgen re-administration for 4 days (C, n = 4) or 2 weeks (D, n = 5). Deletion status of *Klf5* is also shown.
(TIF)
