# Supplementary figures and images for: Different Expression Patterns and Functions of Acetylated and Unacetylated Klf5 in the Proliferation and Differentiation of Prostatic Epithelial Cells
Source: PLoS One. 2013 Jun 5;8(6):e65538. doi: 10.1371/journal.pone.0065538 (PMC3673967; doi:10.1371/journal.pone.0065538)

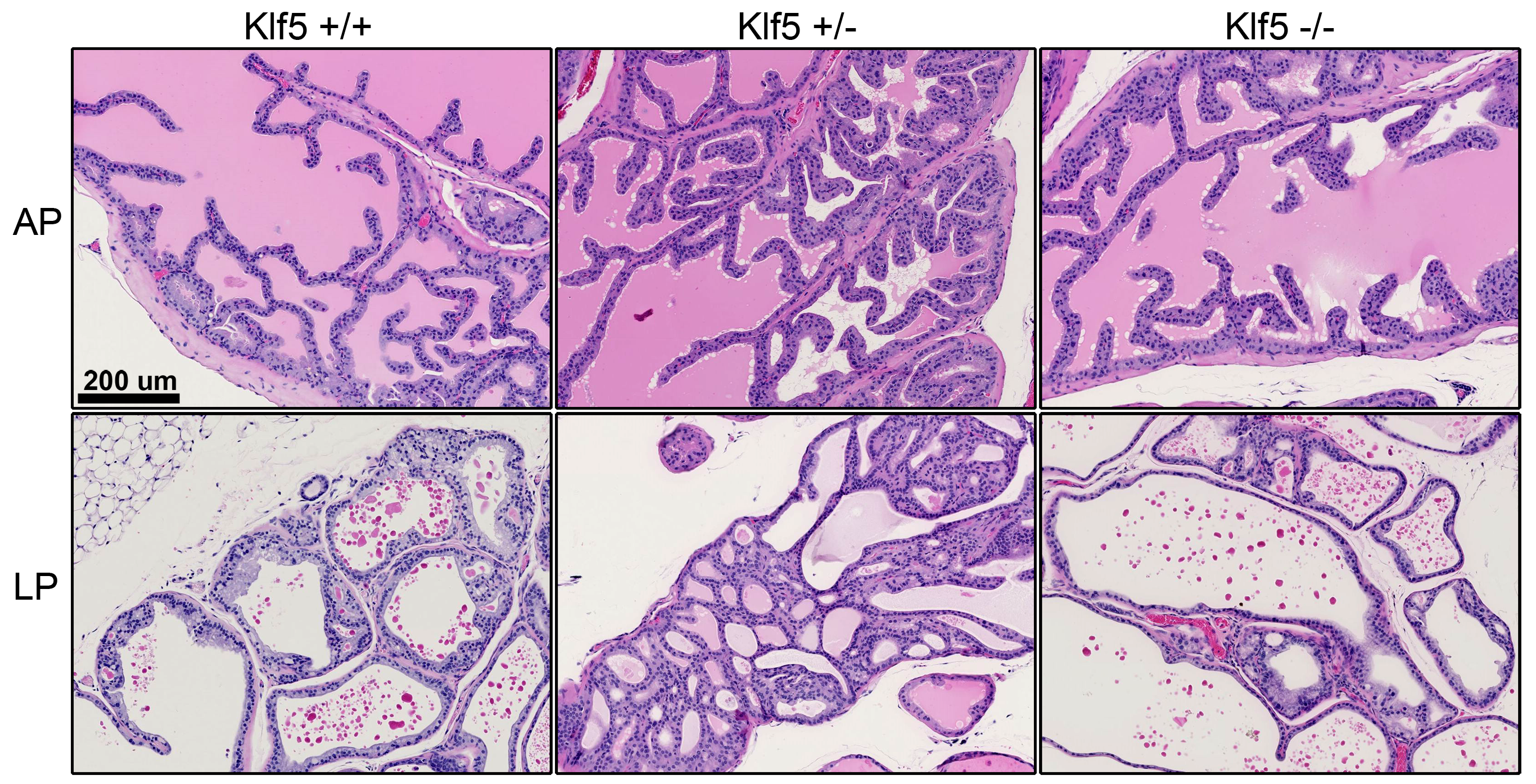

Supplement: Figure S1 — Lower magnification (40X) views of anterior and lateral prostates with different status of Klf5 deletion. Architectural differences are more obvious in these two lobes of the prostate at lower magnification. Prostates from 2-year-old mice with different deletion status of Klf5 were subjected to H&E staining and histological analysis. (TIF) [file pone.0065538.s001.tif]

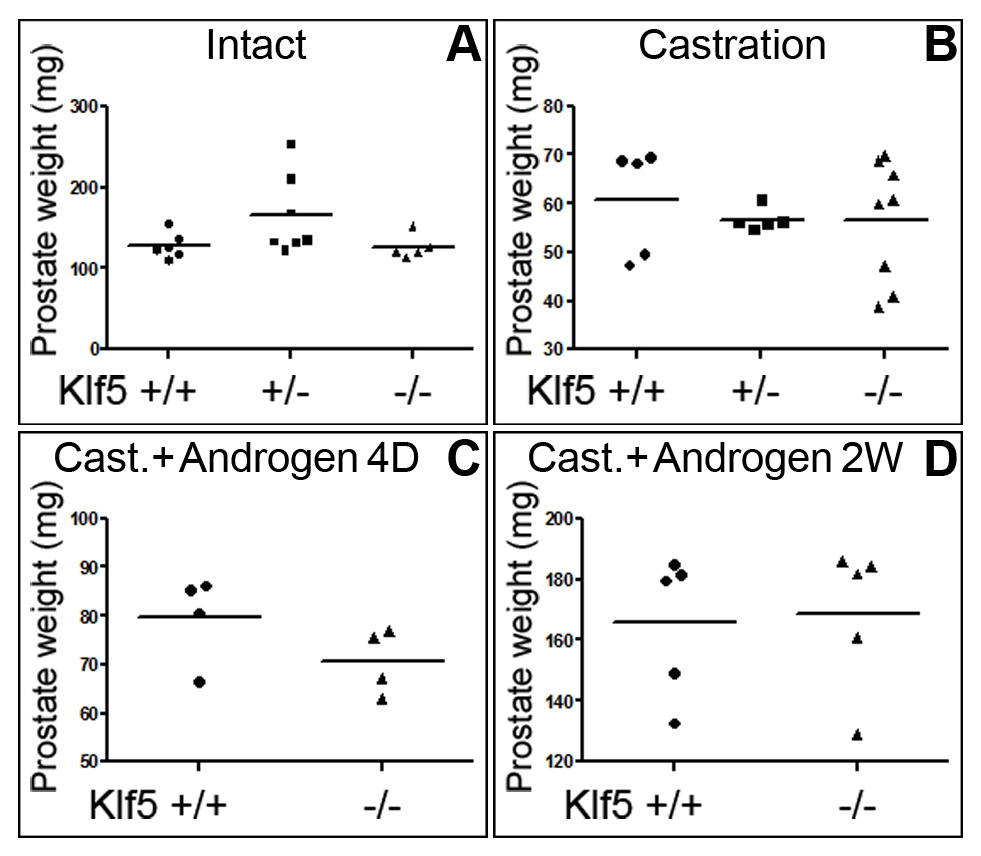

Supplement: Figure S2 — Effects of androgen ablation on prostate weights with different status of Klf5 deletion. A. Weights of prostates with wildtype Klf5 (+/+), hemizygous deletion of Klf5, or homozygous deletion of Klf5 from 21-month-old intact mice without the ablation of androgen (n> = 5). B. Weights of prostates after 5 weeks of androgen ablation from 21-month-old mice (n> = 5). C & D. Weights of prostates from adult mice that underwent castration for 5 weeks and subsequent androgen re-administration for 4 days (C, n = 4) or 2 weeks (D, n = 5). Deletion status of Klf5 is also shown. (TIF) [file pone.0065538.s002.tif]
